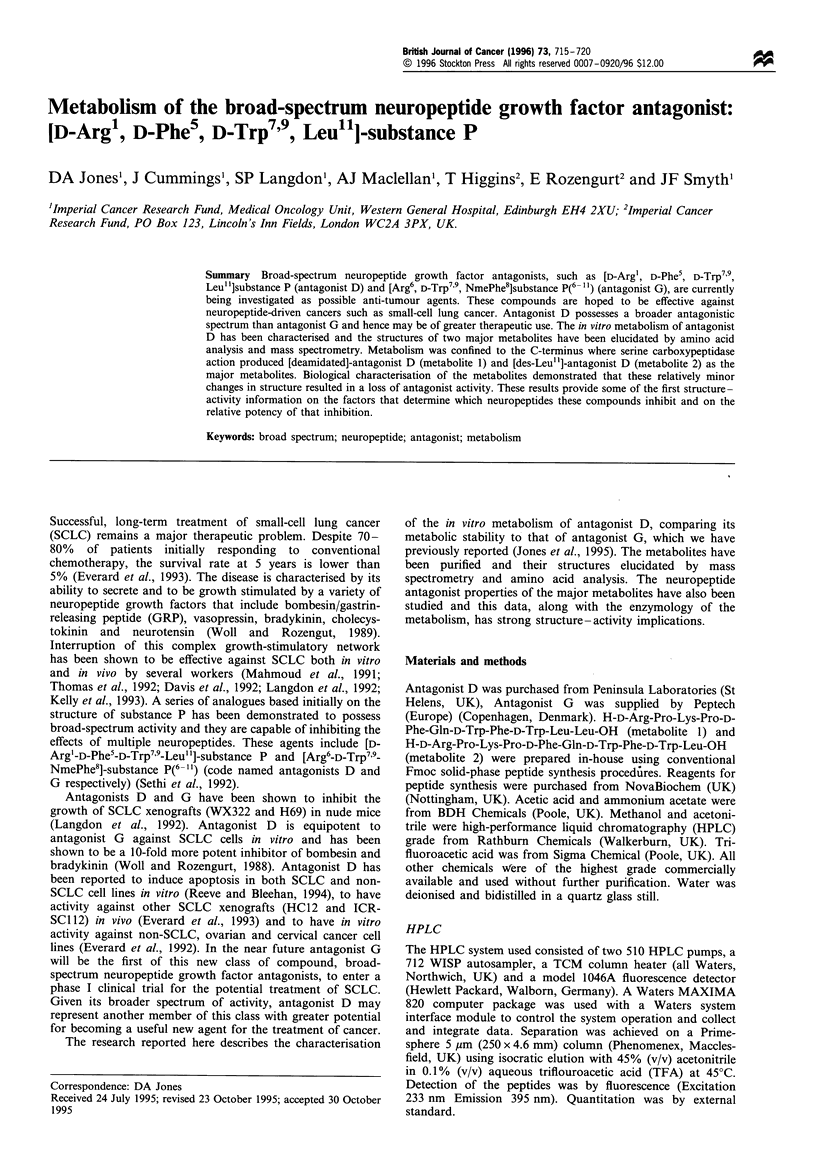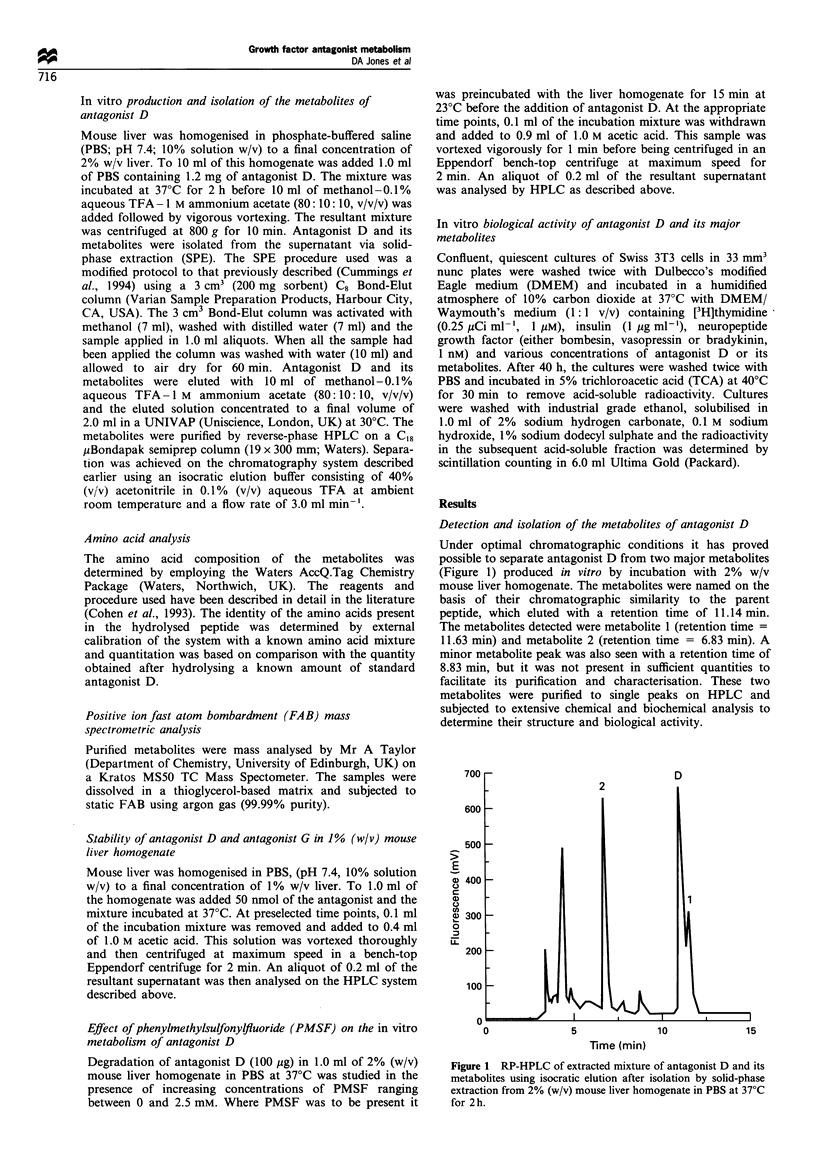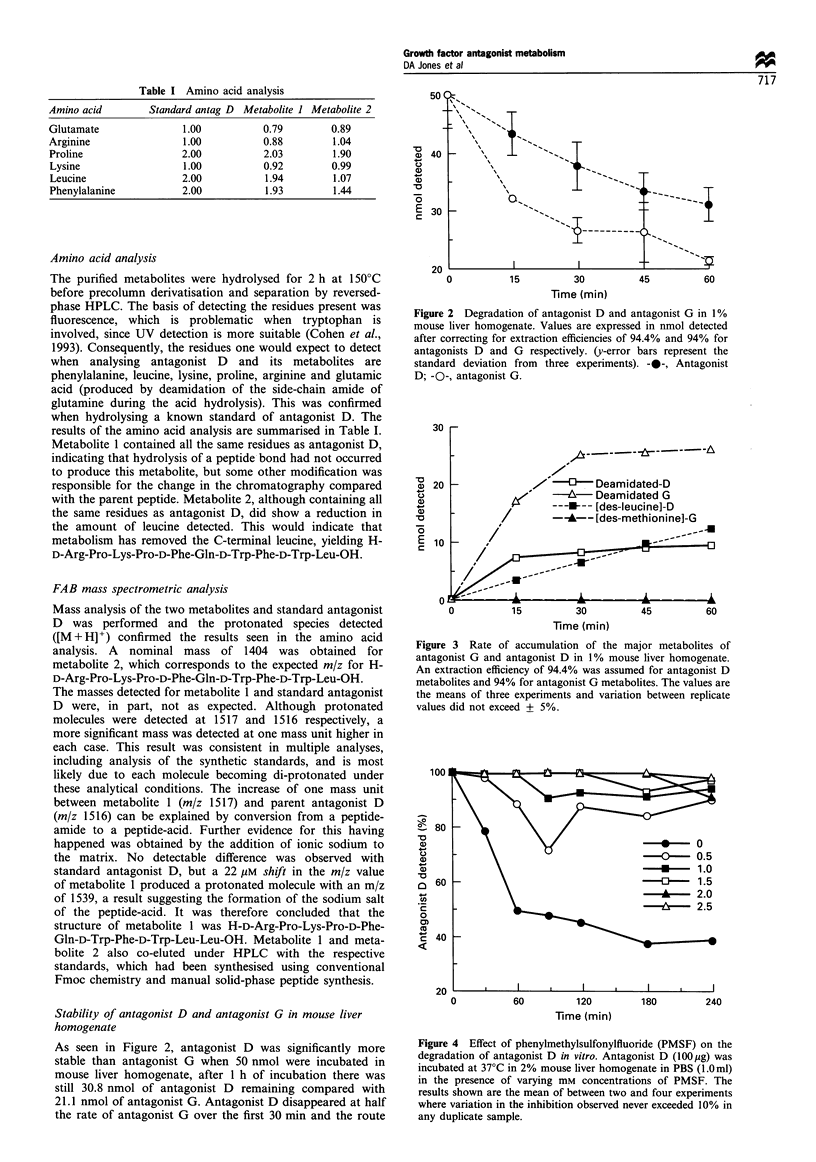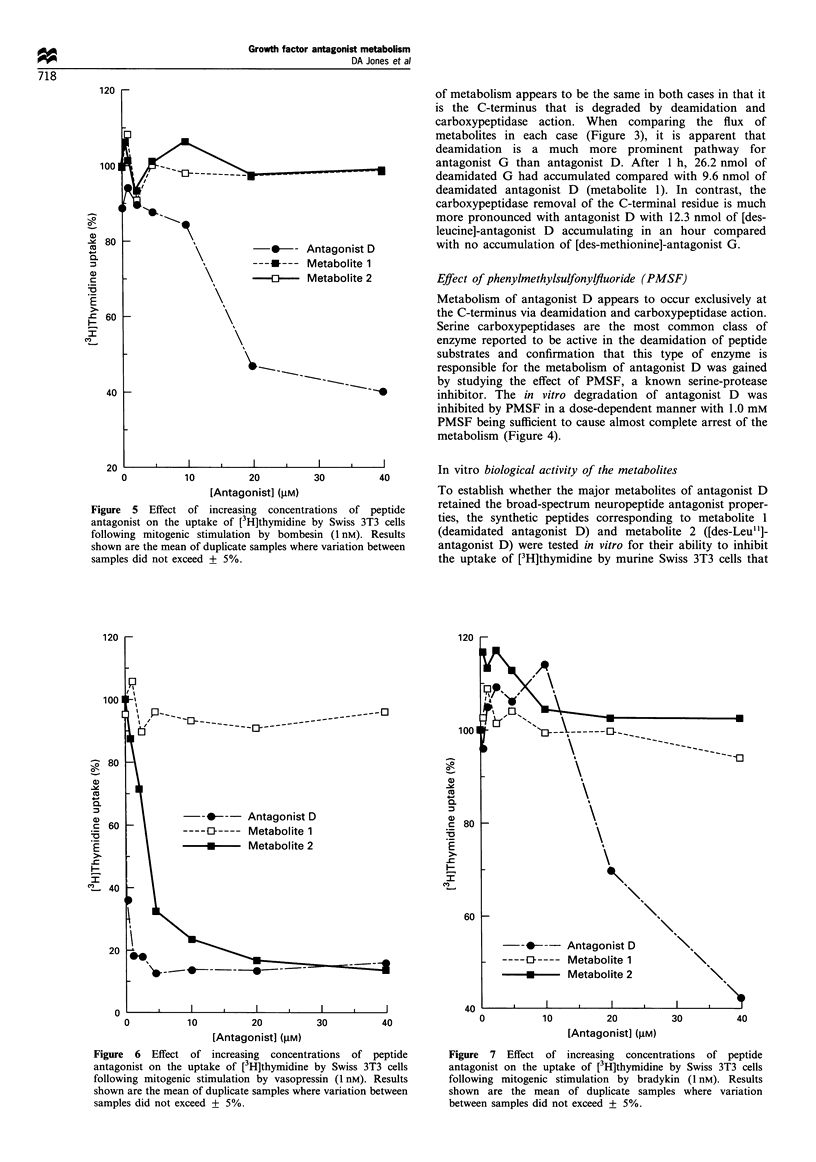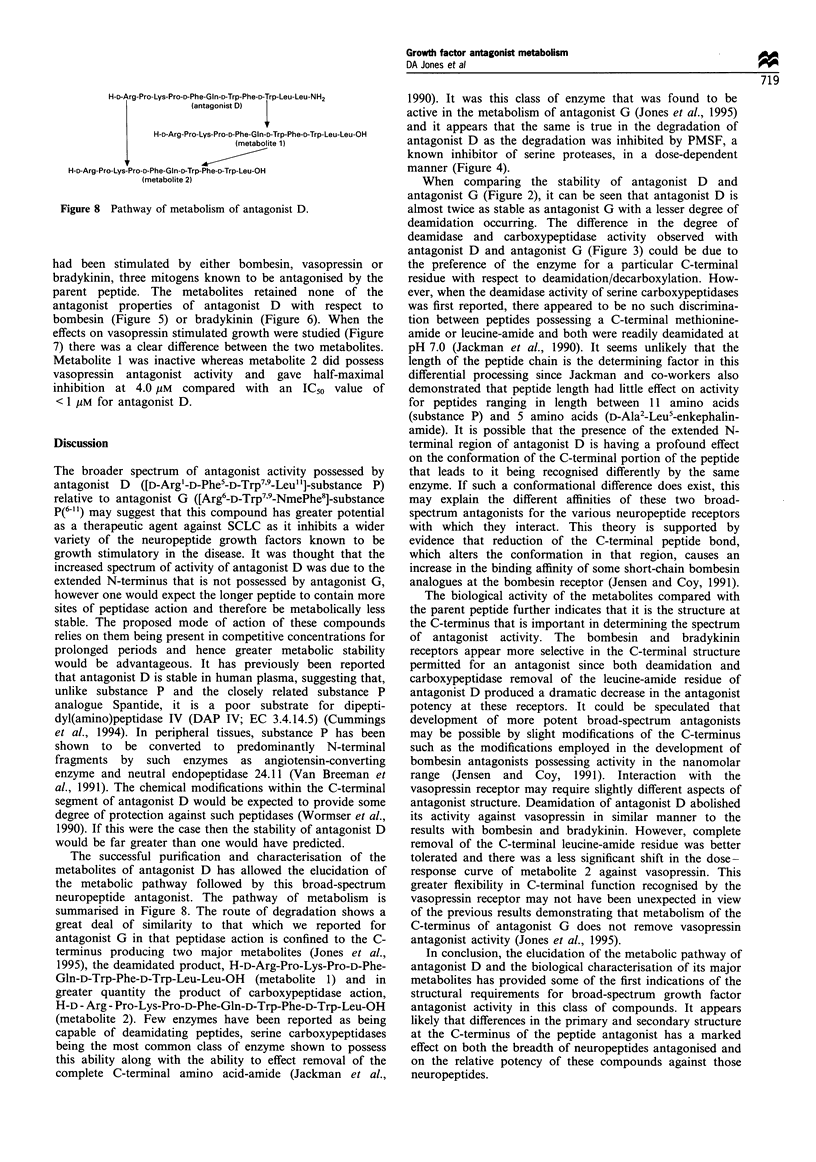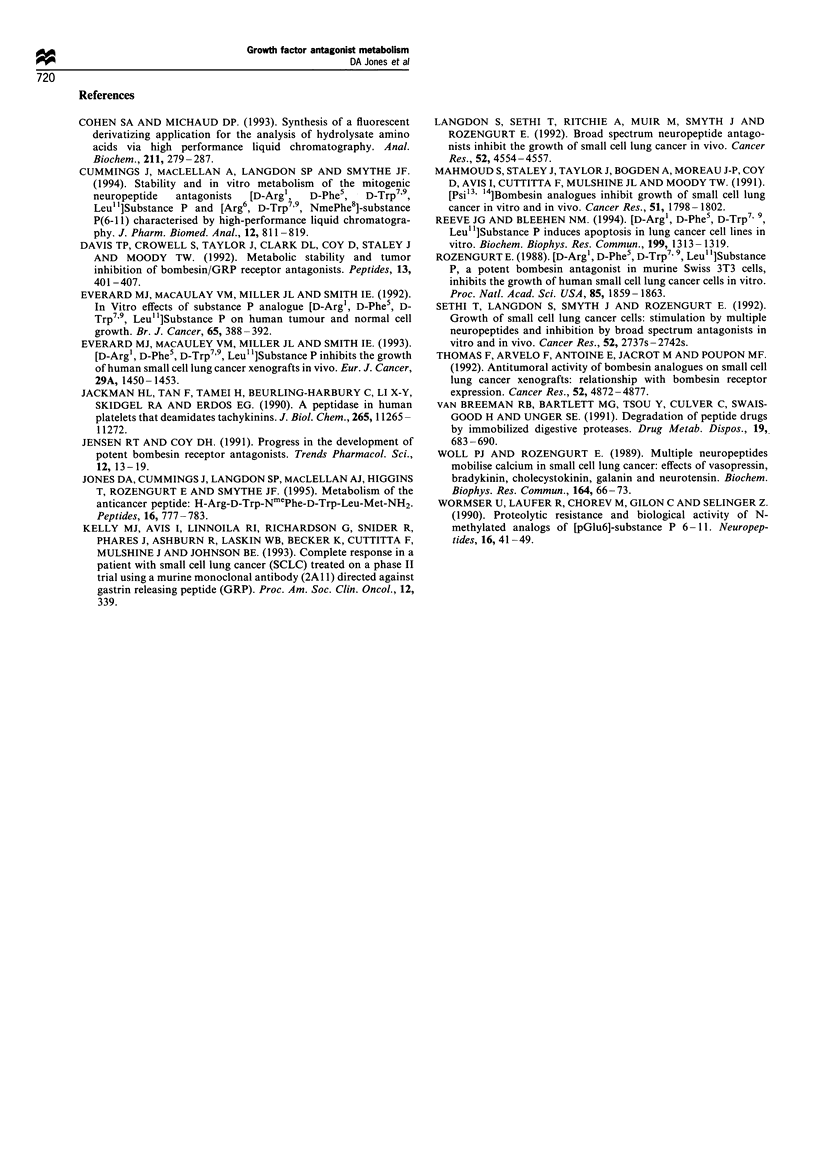# Metabolism of the broad-spectrum neuropeptide growth factor antagonist: [D-Arg1, D-Phe5, D-Trp7,9, Leu11]-substance P.

**DOI:** 10.1038/bjc.1996.126

**Published:** 1996-03

**Authors:** D. A. Jones, J. Cummings, S. P. Langdon, A. J. Maclellan, T. Higgins, E. Rozengurt, J. F. Smyth

**Affiliations:** Medical Oncology Unit, Western General Hospital, Edinburg, UK.

## Abstract

Broad-spectrum neuropeptide growth factor antagonists, such as [D-Arg1, D-Phe5, D-Trp7,9, Leu11]substance P (antagonist D) and [Arg6, D-Trp7,9, NmePhe8]substance P(6-11) (antagonist G), are currently being investigated as possible anti-tumour agents. These compounds are hoped to be effective against neuropeptide-driven cancers such as small-cell lung cancer. Antagonist D possesses a broader antagonistic spectrum than antagonist G and hence may be of greater therapeutic use. The in vitro metabolism of antagonist D has been characterised and the structures of two major metabolites have been elucidated by amino acid analysis and mass spectrometry. Metabolism was confined to the C-terminus where serine carboxypeptidase action produced [deamidated]-antagonist D (metabolite 1) and [des-Leu11]-antagonist D (metabolite 2) as the major metabolites. Biological characterisation of the metabolites demonstrated that these relatively minor changes in structure resulted in a loss of antagonist activity. These results provide some of the first structure-activity information on the factors that determine which neuropeptides these compounds inhibit and on the relative potency of that inhibition.


British Journal of Cancer (1996) 73, 715-720
© 1996 Stockton Press All rights reserved 0007-0920/96 $12.00

### Metabolism of the broad-spectrum neuropeptide growth factor antagonist: [D-Arg$^1$, D-Phe$^5$, D-Trp$^{7,9}$, Leu$^{11}$]-substance P

DA Jones[1], J Cummings[1], SP Langdon[1], AJ Maclellan[1], T Higgins[2], E Rozengurt[2] and JF Smyth[1]

[1]*Imperial Cancer Research Fund, Medical Oncology Unit, Western General Hospital, Edinburgh EH4 2XU;* [2]*Imperial Cancer Research Fund, PO Box 123, Lincoln's Inn Fields, London WC2A 3PX, UK.*

**Summary** Broad-spectrum neuropeptide growth factor antagonists, such as [D-Arg$^1$, D-Phe$^5$, D-Trp$^{7,9}$, Leu$^{11}$]substance P (antagonist D) and [Arg$^6$, D-Trp$^{7,9}$, NmePhe$^8$]substance P($^{6-11}$) (antagonist G), are currently being investigated as possible anti-tumour agents. These compounds are hoped to be effective against neuropeptide-driven cancers such as small-cell lung cancer. Antagonist D possesses a broader antagonistic spectrum than antagonist G and hence may be of greater therapeutic use. The *in vitro* metabolism of antagonist D has been characterised and the structures of two major metabolites have been elucidated by amino acid analysis and mass spectrometry. Metabolism was confined to the C-terminus where serine carboxypeptidase action produced [deamidated]-antagonist D (metabolite 1) and [des-Leu$^{11}$]-antagonist D (metabolite 2) as the major metabolites. Biological characterisation of the metabolites demonstrated that these relatively minor changes in structure resulted in a loss of antagonist activity. These results provide some of the first structure-activity information on the factors that determine which neuropeptides these compounds inhibit and on the relative potency of that inhibition.

**Keywords:** broad spectrum; neuropeptide; antagonist; metabolism

Successful, long-term treatment of small-cell lung cancer (SCLC) remains a major therapeutic problem. Despite 70-80% of patients initially responding to conventional chemotherapy, the survival rate at 5 years is lower than 5% (Everard *et al.*, 1993). The disease is characterised by its ability to secrete and to be growth stimulated by a variety of neuropeptide growth factors that include bombesin/gastrin-releasing peptide (GRP), vasopressin, bradykinin, cholecystokinin and neurotensin (Woll and Rozengut, 1989). Interruption of this complex growth-stimulatory network has been shown to be effective against SCLC both *in vitro* and *in vivo* by several workers (Mahmoud *et al.*, 1991; Thomas *et al.*, 1992; Davis *et al.*, 1992; Langdon *et al.*, 1992; Kelly *et al.*, 1993). A series of analogues based initially on the structure of substance P has been demonstrated to possess broad-spectrum activity and they are capable of inhibiting the effects of multiple neuropeptides. These agents include [D-Arg$^1$-D-Phe$^5$-D-Trp$^{7,9}$-Leu$^{11}$]-substance P and [Arg$^6$-D-Trp$^{7,9}$-NmePhe$^8$]-substance P($^{6-11}$) (code named antagonists D and G respectively) (Sethi *et al.*, 1992).

Antagonists D and G have been shown to inhibit the growth of SCLC xenografts (WX322 and H69) in nude mice (Langdon *et al.*, 1992). Antagonist D is equipotent to antagonist G against SCLC cells *in vitro* and has been shown to be a 10-fold more potent inhibitor of bombesin and bradykinin (Woll and Rozengurt, 1988). Antagonist D has been reported to induce apoptosis in both SCLC and non-SCLC cell lines *in vitro* (Reeve and Bleehan, 1994), to have activity against other SCLC xenografts (HC12 and ICR-SC112) *in vivo* (Everard *et al.*, 1993) and to have *in vitro* activity against non-SCLC, ovarian and cervical cancer cell lines (Everard *et al.*, 1992). In the near future antagonist G will be the first of this new class of compound, broad-spectrum neuropeptide growth factor antagonists, to enter a phase I clinical trial for the potential treatment of SCLC. Given its broader spectrum of activity, antagonist D may represent another member of this class with greater potential for becoming a useful new agent for the treatment of cancer.

The research reported here describes the characterisation of the *in vitro* metabolism of antagonist D, comparing its metabolic stability to that of antagonist G, which we have previously reported (Jones *et al.*, 1995). The metabolites have been purified and their structures elucidated by mass spectrometry and amino acid analysis. The neuropeptide antagonist properties of the major metabolites have also been studied and this data, along with the enzymology of the metabolism, has strong structure-activity implications.

### Materials and methods

Antagonist D was purchased from Peninsula Laboratories (St Helens, UK), Antagonist G was supplied by Peptech (Europe) (Copenhagen, Denmark). H-D-Arg-Pro-Lys-Pro-D-Phe-Gln-D-Trp-Phe-D-Trp-Leu-Leu-OH (metabolite 1) and H-D-Arg-Pro-Lys-Pro-D-Phe-Gln-D-Trp-Phe-D-Trp-Leu-OH (metabolite 2) were prepared in-house using conventional Fmoc solid-phase peptide synthesis procedures. Reagents for peptide synthesis were purchased from NovaBiochem (UK) (Nottingham, UK). Acetic acid and ammonium acetate were from BDH Chemicals (Poole, UK). Methanol and acetonitrile were high-performance liquid chromatography (HPLC) grade from Rathburn Chemicals (Walkerburn, UK). Trifluoroacetic acid was from Sigma Chemical (Poole, UK). All other chemicals were of the highest grade commercially available and used without further purification. Water was deionised and bidistilled in a quartz glass still.

### *HPLC*

The HPLC system used consisted of two 510 HPLC pumps, a 712 WISP autosampler, a TCM column heater (all Waters, Northwich, UK) and a model 1046A fluorescence detector (Hewlett Packard, Walborn, Germany). A Waters MAXIMA 820 computer package was used with a Waters system interface module to control the system operation and collect and integrate data. Separation was achieved on a Primesphere 5 $\mu$m (250 × 4.6 mm) column (Phenomenex, Macclesfield, UK) using isocratic elution with 45% (v/v) acetonitrile in 0.1% (v/v) aqueous triflouroacetic acid (TFA) at 45°C. Detection of the peptides was by fluorescence (Excitation 233 nm Emission 395 nm). Quantitation was by external standard.

Correspondence: DA Jones
Received 24 July 1995; revised 23 October 1995; accepted 30 October 1995

*In vitro production and isolation of the metabolites of antagonist D*

Mouse liver was homogenised in phosphate-buffered saline (PBS; pH 7.4; 10% solution w/v) to a final concentration of 2% w/v liver. To 10 ml of this homogenate was added 1.0 ml of PBS containing 1.2 mg of antagonist D. The mixture was incubated at 37°C for 2 h before 10 ml of methanol–0.1% aqueous TFA – 1 M ammonium acetate (80 : 10 : 10, v/v/v) was added followed by vigorous vortexing. The resultant mixture was centrifuged at 800 *g* for 10 min. Antagonist D and its metabolites were isolated from the supernatant via solid-phase extraction (SPE). The SPE procedure used was a modified protocol to that previously described (Cummings *et al.*, 1994) using a 3 $cm^3$ (200 mg sorbent) $C_8$ Bond-Elut column (Varian Sample Preparation Products, Harbour City, CA, USA). The 3 $cm^3$ Bond-Elut column was activated with methanol (7 ml), washed with distilled water (7 ml) and the sample applied in 1.0 ml aliquots. When all the sample had been applied the column was washed with water (10 ml) and allowed to air dry for 60 min. Antagonist D and its metabolites were eluted with 10 ml of methanol–0.1% aqueous TFA – 1 M ammonium acetate (80 : 10 : 10, v/v/v) and the eluted solution concentrated to a final volume of 2.0 ml in a UNIVAP (Uniscience, London, UK) at 30°C. The metabolites were purified by reverse-phase HPLC on a $C_{18}$ $\mu$Bondapak semiprep column (19 × 300 mm; Waters). Separation was achieved on the chromatography system described earlier using an isocratic elution buffer consisting of 40% (v/v) acetonitrile in 0.1% (v/v) aqueous TFA at ambient room temperature and a flow rate of 3.0 ml $min^{-1}$.

*Amino acid analysis*

The amino acid composition of the metabolites was determined by employing the Waters AccQ.Tag Chemistry Package (Waters, Northwich, UK). The reagents and procedure used have been described in detail in the literature (Cohen *et al.*, 1993). The identity of the amino acids present in the hydrolysed peptide was determined by external calibration of the system with a known amino acid mixture and quantitation was based on comparison with the quantity obtained after hydrolysing a known amount of standard antagonist D.

*Positive ion fast atom bombardment (FAB) mass spectrometric analysis*

Purified metabolites were mass analysed by Mr A Taylor (Department of Chemistry, University of Edinburgh, UK) on a Kratos MS50 TC Mass Spectometer. The samples were dissolved in a thioglycerol-based matrix and subjected to static FAB using argon gas (99.99% purity).

*Stability of antagonist D and antagonist G in 1% (w/v) mouse liver homogenate*

Mouse liver was homogenised in PBS, (pH 7.4, 10% solution w/v) to a final concentration of 1% w/v liver. To 1.0 ml of the homogenate was added 50 nmol of the antagonist and the mixture incubated at 37°C. At preselected time points, 0.1 ml of the incubation mixture was removed and added to 0.4 ml of 1.0 M acetic acid. This solution was vortexed thoroughly and then centrifuged at maximum speed in a bench-top Eppendorf centrifuge for 2 min. An aliquot of 0.2 ml of the resultant supernatant was then analysed on the HPLC system described above.

*Effect of phenylmethylsulfonylfluoride (PMSF) on the in vitro metabolism of antagonist D*

Degradation of antagonist D (100 $\mu$g) in 1.0 ml of 2% (w/v) mouse liver homogenate in PBS at 37°C was studied in the presence of increasing concentrations of PMSF ranging between 0 and 2.5 mM. Where PMSF was to be present it was preincubated with the liver homogenate for 15 min at 23°C before the addition of antagonist D. At the appropriate time points, 0.1 ml of the incubation mixture was withdrawn and added to 0.9 ml of 1.0 M acetic acid. This sample was vortexed vigorously for 1 min before being centrifuged in an Eppendorf bench-top centrifuge at maximum speed for 2 min. An aliquot of 0.2 ml of the resultant supernatant was analysed by HPLC as described above.

*In vitro biological activity of antagonist D and its major metabolites*

Confluent, quiescent cultures of Swiss 3T3 cells in 33 $mm^3$ nunc plates were washed twice with Dulbecco's modified Eagle medium (DMEM) and incubated in a humidified atmosphere of 10% carbon dioxide at 37°C with DMEM/Waymouth's medium (1 : 1 v/v) containing [$^3$H]thymidine (0.25 $\mu$Ci $ml^{-1}$, 1 $\mu$M), insulin (1 $\mu$g $ml^{-1}$), neuropeptide growth factor (either bombesin, vasopressin or bradykinin, 1 nM) and various concentrations of antagonist D or its metabolites. After 40 h, the cultures were washed twice with PBS and incubated in 5% trichloroacetic acid (TCA) at 40°C for 30 min to remove acid-soluble radioactivity. Cultures were washed with industrial grade ethanol, solubilised in 1.0 ml of 2% sodium hydrogen carbonate, 0.1 M sodium hydroxide, 1% sodium dodecyl sulphate and the radioactivity in the subsequent acid-soluble fraction was determined by scintillation counting in 6.0 ml Ultima Gold (Packard).

### Results

*Detection and isolation of the metabolites of antagonist D*

Under optimal chromatographic conditions it has proved possible to separate antagonist D from two major metabolites (Figure 1) produced *in vitro* by incubation with 2% w/v mouse liver homogenate. The metabolites were named on the basis of their chromatographic similarity to the parent peptide, which eluted with a retention time of 11.14 min. The metabolites detected were metabolite 1 (retention time = 11.63 min) and metabolite 2 (retention time = 6.83 min). A minor metabolite peak was also seen with a retention time of 8.83 min, but it was not present in sufficient quantities to facilitate its purification and characterisation. These two metabolites were purified to single peaks on HPLC and subjected to extensive chemical and biochemical analysis to determine their structure and biological activity.

**Figure 1** RP-HPLC of extracted mixture of antagonist D and its metabolites using isocratic elution after isolation by solid-phase extraction from 2% (w/v) mouse liver homogenate in PBS at 37°C for 2 h.

Table I Amino acid analysis

| Amino acid | Standard antag D | Metabolite 1 | Metabolite 2 |
|---|---|---|---|
| Glutamate | 1.00 | 0.79 | 0.89 |
| Arginine | 1.00 | 0.88 | 1.04 |
| Proline | 2.00 | 2.03 | 1.90 |
| Lysine | 1.00 | 0.92 | 0.99 |
| Leucine | 2.00 | 1.94 | 1.07 |
| Phenylalanine | 2.00 | 1.93 | 1.44 |

*Amino acid analysis*

The purified metabolites were hydrolysed for 2 h at 150°C before precolumn derivatisation and separation by reversed-phase HPLC. The basis of detecting the residues present was fluorescence, which is problematic when tryptophan is involved, since UV detection is more suitable (Cohen *et al.*, 1993). Consequently, the residues one would expect to detect when analysing antagonist D and its metabolites are phenylalanine, leucine, lysine, proline, arginine and glutamic acid (produced by deamidation of the side-chain amide of glutamine during the acid hydrolysis). This was confirmed when hydrolysing a known standard of antagonist D. The results of the amino acid analysis are summarised in Table I. Metabolite 1 contained all the same residues as antagonist D, indicating that hydrolysis of a peptide bond had not occurred to produce this metabolite, but some other modification was responsible for the change in the chromatography compared with the parent peptide. Metabolite 2, although containing all the same residues as antagonist D, did show a reduction in the amount of leucine detected. This would indicate that metabolism has removed the C-terminal leucine, yielding H-D-Arg-Pro-Lys-Pro-D-Phe-Gln-D-Trp-Phe-D-Trp-Leu-OH.

*FAB mass spectrometric analysis*

Mass analysis of the two metabolites and standard antagonist D was performed and the protonated species detected ($[M+H]^+$) confirmed the results seen in the amino acid analysis. A nominal mass of 1404 was obtained for metabolite 2, which corresponds to the expected *m/z* for H-D-Arg-Pro-Lys-Pro-D-Phe-Gln-D-Trp-Phe-D-Trp-Leu-OH.

The masses detected for metabolite 1 and standard antagonist D were, in part, not as expected. Although protonated molecules were detected at 1517 and 1516 respectively, a more significant mass was detected at one mass unit higher in each case. This result was consistent in multiple analyses, including analysis of the synthetic standards, and is most likely due to each molecule becoming di-protonated under these analytical conditions. The increase of one mass unit between metabolite 1 (*m/z* 1517) and parent antagonist D (*m/z* 1516) can be explained by conversion from a peptide-amide to a peptide-acid. Further evidence for this having happened was obtained by the addition of ionic sodium to the matrix. No detectable difference was observed with standard antagonist D, but a 22 μM *shift* in the *m/z* value of metabolite 1 produced a protonated molecule with an m/z of 1539, a result suggesting the formation of the sodium salt of the peptide-acid. It was therefore concluded that the structure of metabolite 1 was H-D-Arg-Pro-Lys-Pro-D-Phe-Gln-D-Trp-Phe-D-Trp-Leu-Leu-OH. Metabolite 1 and metabolite 2 also co-eluted under HPLC with the respective standards, which had been synthesised using conventional Fmoc chemistry and manual solid-phase peptide synthesis.

*Stability of antagonist D and antagonist G in mouse liver homogenate*

As seen in Figure 2, antagonist D was significantly more stable than antagonist G when 50 nmol were incubated in mouse liver homogenate, after 1 h of incubation there was still 30.8 nmol of antagonist D remaining compared with 21.1 nmol of antagonist G. Antagonist D disappeared at half the rate of antagonist G over the first 30 min and the route

**Figure 2** Degradation of antagonist D and antagonist G in 1% mouse liver homogenate. Values are expressed in nmol detected after correcting for extraction efficiencies of 94.4% and 94% for antagonists D and G respectively. (*y*-error bars represent the standard deviation from three experiments). -●-, Antagonist D; -○-, antagonist G.

**Figure 3** Rate of accumulation of the major metabolites of antagonist G and antagonist D in 1% mouse liver homogenate. An extraction efficiency of 94.4% was assumed for antagonist D metabolites and 94% for antagonist G metabolites. The values are the means of three experiments and variation between replicate values did not exceed ± 5%.

**Figure 4** Effect of phenylmethylsulfonylfluoride (PMSF) on the degradation of antagonist D *in vitro*. Antagonist D (100 μg) was incubated at 37°C in 2% mouse liver homogenate in PBS (1.0 ml) in the presence of varying mM concentrations of PMSF. The results shown are the mean of between two and four experiments where variation in the inhibition observed never exceeded 10% in any duplicate sample.

**Figure 5** Effect of increasing concentrations of peptide antagonist on the uptake of [$^3$H]thymidine by Swiss 3T3 cells following mitogenic stimulation by bombesin (1 nM). Results shown are the mean of duplicate samples where variation between samples did not exceed ± 5%.

of metabolism appears to be the same in both cases in that it is the C-terminus that is degraded by deamidation and carboxypeptidase action. When comparing the flux of metabolites in each case (Figure 3), it is apparent that deamidation is a much more prominent pathway for antagonist G than antagonist D. After 1 h, 26.2 nmol of deamidated G had accumulated compared with 9.6 nmol of deamidated antagonist D (metabolite 1). In contrast, the carboxypeptidase removal of the C-terminal residue is much more pronounced with antagonist D with 12.3 nmol of [des-leucine]-antagonist D accumulating in an hour compared with no accumulation of [des-methionine]-antagonist G.

*Effect of phenylmethylsulfonylfluoride (PMSF)*

Metabolism of antagonist D appears to occur exclusively at the C-terminus via deamidation and carboxypeptidase action. Serine carboxypeptidases are the most common class of enzyme reported to be active in the deamidation of peptide substrates and confirmation that this type of enzyme is responsible for the metabolism of antagonist D was gained by studying the effect of PMSF, a known serine-protease inhibitor. The *in vitro* degradation of antagonist D was inhibited by PMSF in a dose-dependent manner with 1.0 mM PMSF being sufficient to cause almost complete arrest of the metabolism (Figure 4).

In vitro *biological activity of the metabolites*

To establish whether the major metabolites of antagonist D retained the broad-spectrum neuropeptide antagonist properties, the synthetic peptides corresponding to metabolite 1 (deamidated antagonist D) and metabolite 2 ([des-Leu[11]]-antagonist D) were tested *in vitro* for their ability to inhibit the uptake of [$^3$H]thymidine by murine Swiss 3T3 cells that

**Figure 6** Effect of increasing concentrations of peptide antagonist on the uptake of [$^3$H]thymidine by Swiss 3T3 cells following mitogenic stimulation by vasopressin (1 nM). Results shown are the mean of duplicate samples where variation between samples did not exceed ± 5%.

**Figure 7** Effect of increasing concentrations of peptide antagonist on the uptake of [$^3$H]thymidine by Swiss 3T3 cells following mitogenic stimulation by bradykin (1 nM). Results shown are the mean of duplicate samples where variation between samples did not exceed ± 5%.

**Figure 8** Pathway of metabolism of antagonist D.

had been stimulated by either bombesin, vasopressin or bradykinin, three mitogens known to be antagonised by the parent peptide. The metabolites retained none of the antagonist properties of antagonist D with respect to bombesin (Figure 5) or bradykinin (Figure 6). When the effects on vasopressin stimulated growth were studied (Figure 7) there was a clear difference between the two metabolites. Metabolite 1 was inactive whereas metabolite 2 did possess vasopressin antagonist activity and gave half-maximal inhibition at 4.0 $\mu$M compared with an $IC_{50}$ value of $<1$ $\mu$M for antagonist D.

### Discussion

The broader spectrum of antagonist activity possessed by antagonist D ([D-Arg$^1$-D-Phe$^5$-D-Trp$^{7,9}$-Leu$^{11}$]-substance P) relative to antagonist G ([Arg$^6$-D-Trp$^{7,9}$-NmePhe$^8$]-substance P$^{(6-11)}$) may suggest that this compound has greater potential as a therapeutic agent against SCLC as it inhibits a wider variety of the neuropeptide growth factors known to be growth stimulatory in the disease. It was thought that the increased spectrum of activity of antagonist D was due to the extended N-terminus that is not possessed by antagonist G, however one would expect the longer peptide to contain more sites of peptidase action and therefore be metabolically less stable. The proposed mode of action of these compounds relies on them being present in competitive concentrations for prolonged periods and hence greater metabolic stability would be advantageous. It has previously been reported that antagonist D is stable in human plasma, suggesting that, unlike substance P and the closely related substance P analogue Spantide, it is a poor substrate for dipeptidyl(amino)peptidase IV (DAP IV; EC 3.4.14.5) (Cummings *et al.*, 1994). In peripheral tissues, substance P has been shown to be converted to predominantly N-terminal fragments by such enzymes as angiotensin-converting enzyme and neutral endopeptidase 24.11 (Van Breeman *et al.*, 1991). The chemical modifications within the C-terminal segment of antagonist D would be expected to provide some degree of protection against such peptidases (Wormser *et al.*, 1990). If this were the case then the stability of antagonist D would be far greater than one would have predicted.

The successful purification and characterisation of the metabolites of antagonist D has allowed the elucidation of the metabolic pathway followed by this broad-spectrum neuropeptide antagonist. The pathway of metabolism is summarised in Figure 8. The route of degradation shows a great deal of similarity to that which we reported for antagonist G in that peptidase action is confined to the C-terminus producing two major metabolites (Jones *et al.*, 1995), the deamidated product, H-D-Arg-Pro-Lys-Pro-D-Phe-Gln-D-Trp-Phe-D-Trp-Leu-Leu-OH (metabolite 1) and in greater quantity the product of carboxypeptidase action, H-D-Arg-Pro-Lys-Pro-D-Phe-Gln-D-Trp-Phe-D-Trp-Leu-OH (metabolite 2). Few enzymes have been reported as being capable of deamidating peptides, serine carboxypeptidases being the most common class of enzyme shown to possess this ability along with the ability to effect removal of the complete C-terminal amino acid-amide (Jackman *et al.*, 1990). It was this class of enzyme that was found to be active in the metabolism of antagonist G (Jones *et al.*, 1995) and it appears that the same is true in the degradation of antagonist D as the degradation was inhibited by PMSF, a known inhibitor of serine proteases, in a dose-dependent manner (Figure 4).

When comparing the stability of antagonist D and antagonist G (Figure 2), it can be seen that antagonist D is almost twice as stable as antagonist G with a lesser degree of deamidation occurring. The difference in the degree of deamidase and carboxypeptidase activity observed with antagonist D and antagonist G (Figure 3) could be due to the preference of the enzyme for a particular C-terminal residue with respect to deamidation/decarboxylation. However, when the deamidase activity of serine carboxypeptidases was first reported, there appeared to be no such discrimination between peptides possessing a C-terminal methionine-amide or leucine-amide and both were readily deamidated at pH 7.0 (Jackman *et al.*, 1990). It seems unlikely that the length of the peptide chain is the determining factor in this differential processing since Jackman and co-workers also demonstrated that peptide length had little effect on activity for peptides ranging in length between 11 amino acids (substance P) and 5 amino acids (D-Ala$^2$-Leu$^5$-enkephalin-amide). It is possible that the presence of the extended N-terminal region of antagonist D is having a profound effect on the conformation of the C-terminal portion of the peptide that leads to it being recognised differently by the same enzyme. If such a conformational difference does exist, this may explain the different affinities of these two broad-spectrum antagonists for the various neuropeptide receptors with which they interact. This theory is supported by evidence that reduction of the C-terminal peptide bond, which alters the conformation in that region, causes an increase in the binding affinity of some short-chain bombesin analogues at the bombesin receptor (Jensen and Coy, 1991).

The biological activity of the metabolites compared with the parent peptide further indicates that it is the structure at the C-terminus that is important in determining the spectrum of antagonist activity. The bombesin and bradykinin receptors appear more selective in the C-terminal structure permitted for an antagonist since both deamidation and carboxypeptidase removal of the leucine-amide residue of antagonist D produced a dramatic decrease in the antagonist potency at these receptors. It could be speculated that development of more potent broad-spectrum antagonists may be possible by slight modifications of the C-terminus such as the modifications employed in the development of bombesin antagonists possessing activity in the nanomolar range (Jensen and Coy, 1991). Interaction with the vasopressin receptor may require slightly different aspects of antagonist structure. Deamidation of antagonist D abolished its activity against vasopressin in similar manner to the results with bombesin and bradykinin. However, complete removal of the C-terminal leucine-amide residue was better tolerated and there was a less significant shift in the dose–response curve of metabolite 2 against vasopressin. This greater flexibility in C-terminal function recognised by the vasopressin receptor may not have been unexpected in view of the previous results demonstrating that metabolism of the C-terminus of antagonist G does not remove vasopressin antagonist activity (Jones *et al.*, 1995).

In conclusion, the elucidation of the metabolic pathway of antagonist D and the biological characterisation of its major metabolites has provided some of the first indications of the structural requirements for broad-spectrum growth factor antagonist activity in this class of compounds. It appears likely that differences in the primary and secondary structure at the C-terminus of the peptide antagonist has a marked effect on both the breadth of neuropeptides antagonised and on the relative potency of these compounds against those neuropeptides.

### References

COHEN SA AND MICHAUD DP. (1993). Synthesis of a fluorescent derivatizing application for the analysis of hydrolysate amino acids via high performance liquid chromatography. *Anal. Biochem.*, **211**, 279-287.

CUMMINGS J, MACLELLAN A, LANGDON SP AND SMYTHE JF. (1994). Stability and in vitro metabolism of the mitogenic neuropeptide antagonists [D-Arg$^1$, D-Phe$^5$, D-Trp$^{7,9}$, Leu$^{11}$]Substance P and [Arg$^6$, D-Trp$^{7,9}$, NmePhe$^8$]-substance P(6-11) characterised by high-performance liquid chromatography. *J. Pharm. Biomed. Anal.*, **12**, 811-819.

DAVIS TP, CROWELL S, TAYLOR J, CLARK DL, COY D, STALEY J AND MOODY TW. (1992). Metabolic stability and tumor inhibition of bombesin/GRP receptor antagonists. *Peptides*, **13**, 401-407.

EVERARD MJ, MACAULAY VM, MILLER JL AND SMITH IE. (1992). In Vitro effects of substance P analogue [D-Arg$^1$, D-Phe$^5$, D-Trp$^{7,9}$, Leu$^{11}$]Substance P on human tumour and normal cell growth. *Br. J. Cancer*, **65**, 388-392.

EVERARD MJ, MACAULEY VM, MILLER JL AND SMITH IE. (1993). [D-Arg$^1$, D-Phe$^5$, D-Trp$^{7,9}$, Leu$^{11}$]Substance P inhibits the growth of human small cell lung cancer xenografts in vivo. *Eur. J. Cancer*, **29A**, 1450-1453.

JACKMAN HL, TAN F, TAMEI H, BEURLING-HARBURY C, LI X-Y, SKIDGEL RA AND ERDOS EG. (1990). A peptidase in human platelets that deamidates tachykinins. *J. Biol. Chem.*, **265**, 11265-11272.

JENSEN RT AND COY DH. (1991). Progress in the development of potent bombesin receptor antagonists. *Trends Pharmacol. Sci.*, **12**, 13-19.

JONES DA, CUMMINGS J, LANGDON SP, MACLELLAN AJ, HIGGINS T, ROZENGURT E AND SMYTHE JF. (1995). Metabolism of the anticancer peptide: H-Arg-D-Trp-N$^{me}$Phe-D-Trp-Leu-Met-NH$_2$. *Peptides*, **16**, 777-783.

KELLY MJ, AVIS I, LINNOILA RI, RICHARDSON G, SNIDER R, PHARES J, ASHBURN R, LASKIN WB, BECKER K, CUTTITTA F, MULSHINE J AND JOHNSON BE. (1993). Complete response in a patient with small cell lung cancer (SCLC) treated on a phase II trial using a murine monoclonal antibody (2A11) directed against gastrin releasing peptide (GRP). *Proc. Am. Soc. Clin. Oncol.*, **12**, 339.

LANGDON S, SETHI T, RITCHIE A, MUIR M, SMYTH J AND ROZENGURT E. (1992). Broad spectrum neuropeptide antagonists inhibit the growth of small cell lung cancer in vivo. *Cancer Res.*, **52**, 4554-4557.

MAHMOUD S, STALEY J, TAYLOR J, BOGDEN A, MOREAU J-P, COY D, AVIS I, CUTTITTA F, MULSHINE JL AND MOODY TW. (1991). [Psi$^{13,\ 14}$]Bombesin analogues inhibit growth of small cell lung cancer in vitro and in vivo. *Cancer Res.*, **51**, 1798-1802.

REEVE JG AND BLEEHEN NM. (1994). [D-Arg$^1$, D-Phe$^5$, D-Trp$^{7,\ 9}$, Leu$^{11}$]Substance P induces apoptosis in lung cancer cell lines in vitro. *Biochem. Biophys. Res. Commun.*, **199**, 1313-1319.

ROZENGURT E. (1988). [D-Arg$^1$, D-Phe$^5$, D-Trp$^{7,\ 9}$, Leu$^{11}$]Substance P, a potent bombesin antagonist in murine Swiss 3T3 cells, inhibits the growth of human small cell lung cancer cells in vitro. *Proc. Natl. Acad. Sci. USA*, **85**, 1859-1863.

SETHI T, LANGDON S, SMYTH J AND ROZENGURT E. (1992). Growth of small cell lung cancer cells: stimulation by multiple neuropeptides and inhibition by broad spectrum antagonists in vitro and in vivo. *Cancer Res.*, **52**, 2737s-2742s.

THOMAS F, ARVELO F, ANTOINE E, JACROT M AND POUPON MF. (1992). Antitumoral activity of bombesin analogues on small cell lung cancer xenografts: relationship with bombesin receptor expression. *Cancer Res.*, **52**, 4872-4877.

VAN BREEMAN RB, BARTLETT MG, TSOU Y, CULVER C, SWAISGOOD H AND UNGER SE. (1991). Degradation of peptide drugs by immobilized digestive proteases. *Drug Metab. Dispos.*, **19**, 683-690.

WOLL PJ AND ROZENGURT E. (1989). Multiple neuropeptides mobilise calcium in small cell lung cancer: effects of vasopressin, bradykinin, cholecystokinin, galanin and neurotensin. *Biochem. Biophys. Res. Commun.*, **164**, 66-73.

WORMSER U, LAUFER R, CHOREV M, GILON C AND SELINGER Z. (1990). Proteolytic resistance and biological activity of N-methylated analogs of [pGlu6]-substance P 6-11. *Neuropeptides*, **16**, 41-49.